# A novel conjugal donor strain for improved DNA transfer into *Clostridium* spp.

**DOI:** 10.1016/j.anaerobe.2019.06.020

**Published:** 2019-10

**Authors:** Craig Woods, Christopher M. Humphreys, Raquel Mesquita Rodrigues, Patrick Ingle, Peter Rowe, Anne M. Henstra, Michael Köpke, Sean D. Simpson, Klaus Winzer, Nigel P. Minton

**Affiliations:** a Clostridia Research Group, BBSRC/EPSRC Synthetic Biology Research Centre (SBRC), School of Life Sciences, The University of Nottingham, Nottingham, NG7 2RD, UK; b LanzaTech Inc., 8045 Lamon Avenue, Suite 400, Skokie, IL, USA; c NIHR Nottingham Biomedical Research Centre, Nottingham University Hospitals NHS Trust and the University of Nottingham, Nottingham NG7 2RD, UK

**Keywords:** *Clostridium*, DNA transfer, Conjugation, Restriction-methylation systems, *C. difficile*, *C. sporogenes*, *C. autoethanogenum*

## Abstract

*Clostridium* encompasses species which are relevant to human and animal disease as well as species which have industrial potential, for instance, as producers of chemicals and fuels or as tumour delivery vehicles. Genetic manipulation of these target organisms is critical for advances in these fields. DNA transfer efficiencies, however, vary between species. Low efficiencies can impede the progress of research efforts.

A novel conjugal donor strain of *Escherichia coli* has been created which exhibits a greater than 10-fold increases in conjugation efficiency compared to the traditionally used CA434 strain in the three species tested; *C. autoethanogenum* DSM 10061*, C. sporogenes* NCIMB 10696 and *C. difficile* R20291. The novel strain, designated ‘sExpress’, does not methylate DNA at Dcm sites (CCWGG) which allows circumvention of cytosine-specific Type IV restriction systems.

A robust protocol for conjugation is presented which routinely produces in the order of 10^5^ transconjugants per millilitre of donor cells for *C. autoethanogenum*, 10^6^ for *C. sporogenes* and 10^2^ for *C. difficile* R20291. The novel strain created is predicted to be a superior conjugal donor in a wide range of species which possess Type IV restriction systems.

## Highlights

• A novel conjugal donor strain has been constructed to circumvent Type IV restriction.
• Improved DNA transfer to *C. difficile* R20291, *C. sporogenes* and *C. autoethanogenum.*
• A knockout strain of *C. autoethanogenum* exhibited ∼10× higher conjugation efficiency.
• A knockout strain of *C. sporogenes* exhibited ∼10× higher conjugation efficiency.
• Clostron mutant converted to in-frame deletion using CRISPR/Cas9.

## Teaser

Importance: The ability to transfer genetic material into a target organism is crucial for the development of a wide range of targeted genetic manipulation techniques. Overcoming the organisms’ native restriction systems which target foreign incoming DNA is one strategy that can increase the efficiency of genetic transfer. The novel *E. coli* donor strain described here employs this strategy, increasing the frequencies of conjugation into a range of clostridial strains, and therefore opening up the potential to implement novel gene manipulation techniques. Furthermore this novel donor strain has potential applications across a wide range of genetically recalcitrant organisms, and should be beneficial wherever the frequently occurring Type IV restriction systems are possessed by the target in question.

## 1 Introduction

The bacterial genus *Clostridium* is most often associated with disease, none more so than *Clostridium difficile,* the leading cause of antibiotic-associated disease and a significant burden on the finances of healthcare systems worldwide [1]. However, the vast majority of clostridial species are entirely benign and in many instances possess properties and attributes of great benefit to mankind. The acetogen *Clostridium autoethanogenum,* for instance, is the chassis currently being commercialised by LanzaTech for the large-scale conversion of the CO/H_2_-rich waste streams of steel mill off-gas into ethanol for use as a transportation fuel [2]. On the other hand, *Clostridium sporogenes* is being pursued as a vehicle for the delivery of anti-cancer agents to solid tumours [3].

Gene modification and/or bioengineering offer one of the most effective routes to either a better understanding of the molecular pathogenesis of an organism, or to bring about essential improvements to those chassis with commercial potential. Crucial is the ability to efficiently introduce the requisite gene tools into the target clostridial cell. DNA transfer is routinely achieved in *Clostridium* species via either electrotransformation or by conjugative mobilisation from an *Escherichia coli* donor strain [4]. Conjugation is the preferred method of DNA transfer in many species, for instance *Clostridium autoethanogenum* [5]*, Clostridium botulinum* [6]*, Clostridium sporogenes* [7], and *Clostridium difficile* [8]. Nonetheless, in many of these species the frequencies of transfer are relatively low. This is particularly the case with *C. autoethanogenum* and many strains of *C. difficile*, in particular so-called hypervirulent strains belonging to PCR Ribotype 027 [9]. .

There are a variety of *E. coli* strains which are capable of mediating the mobilisation of small, autonomous vectors to clostridial recipients during the process of conjugation. Transfer of the vector generally relies on its *oriT-*mediated mobilisation with the necessary transfer functions being provided *in trans* by genes located either on a plasmid, such as R702, co-resident in the donor *E.coli* host strain (eg., CA434), or in the chromosome, e.g. donor strains S17.1 and SM10 [4]. All genetic studies of *C. autoethanogenum* to date, and the majority of studies in *C. difficile* and *C. sporogenes*, have used the donor *E. coli* strain CA434 [10].

One prominent barrier to transformation is that of host restriction-modification (RM) systems. Restriction systems degrade incoming DNA which lacks the native modifications of the target strain and is hence recognised as ‘foreign’. DNA can be regarded as foreign if an expected modification at a given motif is absent, or where additional modifications which are typically not found in native DNA are present. Type IV restriction systems are characterised as those which recognise foreign modification patterns, whilst Types I-III target unmodified DNA. The three species examined here (*C. autoethanogenum, C, sporogenes* and *C. difficile*) are unified by possessing Type IV restriction systems.

The methylation status of the donor strain has been shown to affect DNA transfer efficiencies in a range of bacterial species including *Bacillus anthracis* [11], *Streptomyces* [12,13], *Bacillus thuringiensis* [14], *Acholeplasma laidlawii* [15], *Streptomyces avermilitis* [16] and *Lactobacillus* [17]. More recently, several studies on clostridia have come to similar conclusions concerning methylation status including *C. thermocellum* DSM 1313 [18], *C. ljungdahlii* DSM 13528 [19], and *C. pasteurianum* NRRL B-598 [20]. In this study the derivation and application of a novel conjugal *E. coli* donor strain designated ‘sExpress’, which lacks Dcm methylation, is described. sExpress offers a significant increase in conjugation efficiency over the previously widely used CA434.

## 2 Results

### 2.1 Restriction systems of *C. autoethanogenum* DSM 10061

The restriction enzyme database http://rebase.neb.com [21] provides an overview of the restriction-modification (RM) systems found in the genome of a given strain. The REBASE profile of *C. autoethanogenum* DSM 10061 predicts five genes to be Type IV restriction enzymes each with an unknown recognition sequence. The attribution of these genes as encoding Type IV systems is due to a shared region of homology with the *E. coli* McrB Type IV cytosine-specific restriction enzyme. McrB is the cytosine-specific binding component of the McrB-McrA complex [22]. *C. autoethanogenum* DSM 10061 is also predicted to possess four methyltransferase genes, one of which is a Type II restriction-modification enzyme (Cau10061II) which has a predicted methylation motif of ‘GTTAAT’. The other three methyltransferases appear to have no associated restriction enzymes and so are not expected to significantly contribute to a restriction barrier. Initially, our research aimed to increase the efficiency of DNA transfer to *C. autoethanogenum* DSM 10061. To achieve this the two most promising approaches appeared to be to generate a knockout of the fused R-M system Cau10061II encoded by CLAU_0514 and to circumvent the effects of the five Type IV system enzymes by removing Dcm methylation from the incoming DNA.

### 2.2 *C. autoethanogenum* ΔCLAU_0514 exhibits an increased conjugation efficiency

**Fig. 1:**
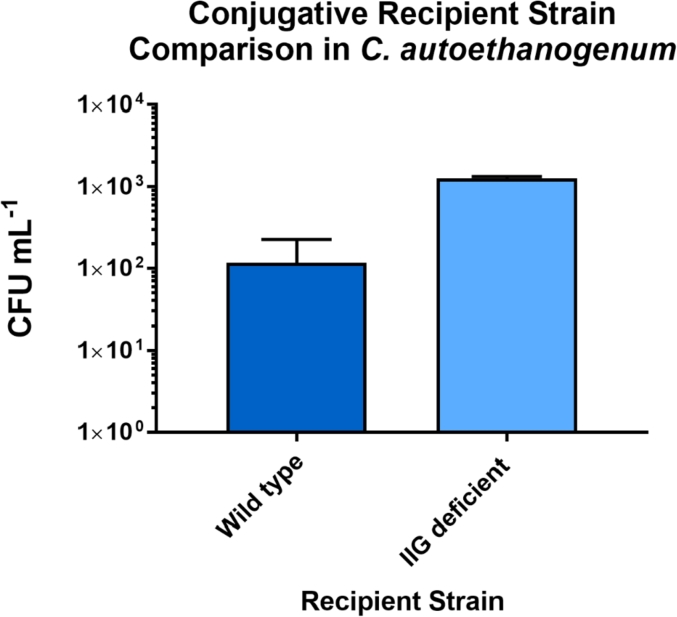
A comparison of conjugation efficiency using either the ΔCLAU_0514 strain of C. autoethanogenum DSM 10061 (IIG deficient) or the wild type strain as the recipient. sExpress was used as a conjugal donor in either case with the only difference in conjugation protocol being the choice of recipient strain. Error bars show the standard deviation. An unpaired two-tailed T-test was performed with Welch's correction to measure the significance of the difference between the two recipient strains giving a P value of <0.0001, n = 4. Four different recipient cultures were used for each condition with optical densities ranging from 0.1 to 0.2. Colony forming units (CFU) ml^−1^ was calculated as the number of colony forming units per ml of recipient culture using 200 μl of conjugal donor culture per conjugation.

CLAU_0514 encodes a fused Type II R-M enzyme which REBASE predicts to have a sequence specificity of GTTAAT. A knockout strain for this gene was created in a wild type *C. autoethanogenum* DSM 10061 background with the aim of creating a strain which is more amenable to DNA transfer. The knockout was generated through CRISPR/Cas9-mediated genome editing [23]. The requisite knock-out plasmid, pMTL43151-CLAU_0514, was constructed as detailed in the methods section. This vector consists of homology arms targeting the CLAU_0514 locus, causing a clean and complete deletion following a double homologous recombination event. The knockout vector was introduced into *C. autoethanogenum* DSM 10061 via conjugation and colonies representing putative mutants were analysed via colony PCR. Sanger sequencing of the colony PCR products was used to confirm a successful deletion. After confirmation of the deletion, a conjugation efficiency experiment was undertaken as detailed in the methods section. The donor strain whose creation is detailed in this text ‘sExpress’ was used to transfer the plasmid pMTL83151 which harbours a thiamphenicol resistance cassette. The newly created ΔCLAU_0514 strain showed a statistically significant (p= <0.0001) increase in the number of transconjugants obtained from conjugation (Fig. 1).

### 2.3 Derivation of the novel strain ‘sExpress’ donor strain

CA434 is essentially *E. coli* HB101 carrying R702, and contains both Dam and Dcm methyltransferases which modify the sequences GATC and CCWGG, respectively. To circumvent the Type IV restriction systems of *C. autoethanogenum* DSM 10061, a strain which does not methylate at Dcm (CCWGG) sites was required. NEB Express (*fhuA2 [lon] ompT gal sulA11 R(mcr-73*:*miniTn10*--*TetS)2 [dcm] R(zgb-210*:*Tn10*--*TetS) endA1 Δ(mcrC-mrr)114*:IS10) is a commercially available strain which fit the requirements to become the basis of our novel conjugal donor, most notably the lack of Dcm methylation. The conjugal transfer functions of CA434 are encoded on the plasmid R702. Since R702 is self-mobilisable, the construction of a novel conjugal donor strain was achieved via conjugative plasmid transfer of R702 from CA434 (donor) to the chosen recipient strain, NEB Express. CA434 carries both a *leuB6* and a *proA2* mutation. R702 confers resistance to tetracycline. CA434 was, therefore, counter-selected on M9 salts minimal medium lacking proline and leucine while transconjugants (NEB Express harbouring R702) were positively selected by the incorporation of tetracycline into the selective media. Transconjugants were re-streaked and the acquisition of the R702 vector confirmed by PCR and subsequent Sanger sequencing. The resulting strain was designated ‘sExpress’.

### 2.4 sExpress is a superior donor strain to CA434 in *C. autoethanogenum* DSM 10061

**Fig. 2:**
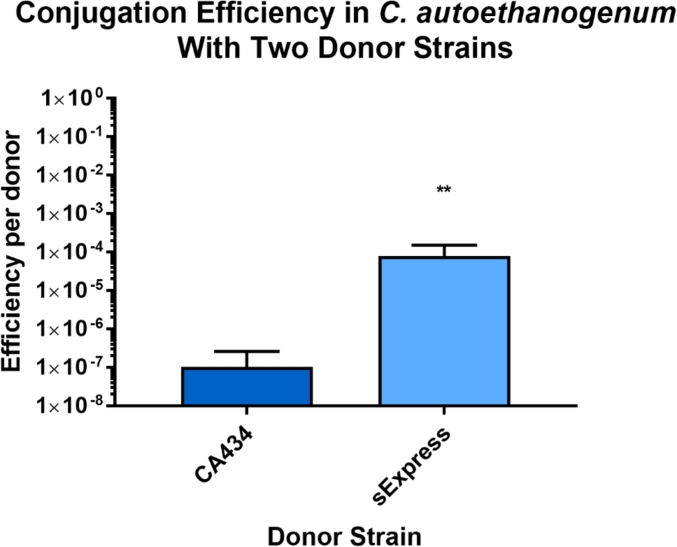
A comparison of conjugation efficiency varying the conjugal donor strain. A single recipient culture was used for both donor strains. Ten separate donor cultures were used in separate conjugations for each donor strain. pMTL83151 was the target plasmid transferred in each case. Error bars represent the standard deviations of each dataset. Two-tailed T-tests using Welch's correction were used to compare the two donor strains (p = 0.0071 **, n = 10).

*C. autoethanogenum* DSM 10061 was used as a recipient in conjugations with the donor strains CA434 or sExpress into which had been introduced the plasmid pMTL83151. This is a modular vector [24] which forms the basis for many vectors commonly used in *C. autoethanogenum*. The plasmid pMTL83151 contains a Gram-positive origin of replication from the *C. butyricum* plasmid pCB102 and the *C. perfringens catP* antibiotic resistance marker that confers resistance to thiamphenicol on clostridial hosts. It was chosen as the representative plasmid to be transferred due to the widespread use of this replicon in genetic studies of *C. autoethanogenum* as well as the generally higher frequencies of transfer which have been noted compared to other Gram-positive origins of replication. It contains nine Dcm sites (CCWGG) which would be methylated in the CA434 strain. The conjugation protocol used to directly compare the two donor strains is detailed in the materials and methods section, with the sole difference being the identity of the donor strain. sExpress produced an average of 9991 (SD = 4782) transconjugants per conjugation whilst CA434 generated an average of 21 (SD = 16). In terms of efficiency per donor cell this represented values of 1.05^−7^ (SD = 1.58^−7^) for CA434 and 7.94^−5^ (SD = 7.23^−5^) for sExpress (Fig. 2).

### 2.5 sExpress in *C. sporogenes*

**Fig. 3:**
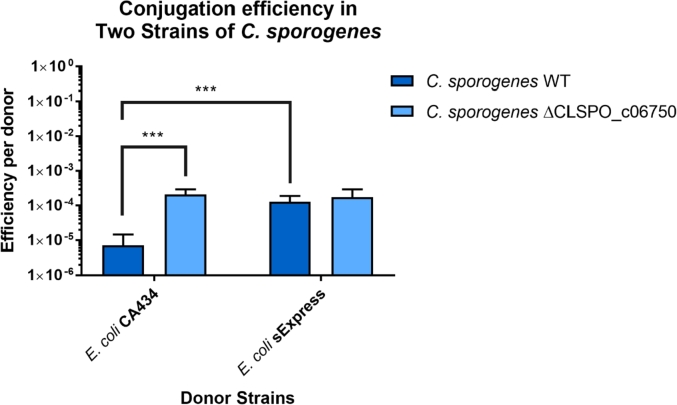
A comparison of conjugation efficiency between two donor strains (CA434 and sExpress) into two different *C. sporogenes* NCIMB 10696 recipient strains. *C. sporogenes* ΔCLSPO_c06750 is a knockout strain for a putative Type IV restriction system. Three recipient strain cultures were grown of both wild type and ΔCLSPO_c06750 and each of these strains was used in three separate conjugations from separate donor strain cultures. This gave a total of nine conjugations for each donor/recipient combination. Conjugation efficiency was calculated as the ratio of CFU on transconjugant selective plates to the CFU of donor cells going into each conjugation. There was a significant difference in conjugation efficiency between CA434 and sExpress in wild type *C. sporogenes* (p = 0.0003 ***, n = 9) and between the wild type and knockout strain when using CA434 (p = 0.0008 ***, n = 9) but not when using sExpress. There was no statistically significance difference in conjugation efficiency between CA434 and sExpress in the ΔCLSPO_c06750 strain (p = 0.0684, n = 9). Two-tailed T-tests using Welch's correction were used in each comparison.

Having established sExpress as a superior conjugal donor strain in conjugations with *C. autoethanogenum*, an analysis of related organisms for which the strain could prove beneficial was undertaken. The presence of a Type IV restriction system, as predicted by REBASE, was indicative that sExpress would be beneficial. One organism highlighted by the analysis was *C. sporogenes* NCIMB 10696 [25]. The transfer frequency into *C. sporogenes* using CA434 was already sufficiently high and the protocol sufficiently robust that standard genetic studies could proceed unhampered. However, if sufficiently high frequencies of transfer could be obtained in this organism then it would be an important step towards implementation of several high throughput techniques such as Multiplex Automated Genomic Engineering (MAGE) [26] or Transposon Directed Insertion Sequencing (TraDIS) [27]. With this in mind, the two donors CA434 and sExpress were compared as for *C. autoethanogenum*. The use of sExpress in *C. sporogenes* NCIMB 10696 improved conjugation efficiencies by approximately one order of magnitude (Fig. 3). To further investigate the effects of Type IV restriction systems on DNA transfer to *C. sporogenes* NCIMB 10696 a knockout strain for a putative Type IV restriction system was created and the conjugation efficiency of both CA434 and sExpress was measured in this strain.

### 2.6 Knocking out the type IV restriction system of *C. sporogenes*

*C. sporogenes* NCIMB 10696 has only a single Type IV restriction enzyme predicted by REBASE. To further investigate the improved transfer when using sExpress as the conjugal donor a knockout strain of *C. sporogenes* NCIMB 10696 for the putative Type IV restriction system gene CLSPO_c06750 encoding Csp10696ORF6750P was generated. According to CD-Search (NCBI), the protein encoded by this gene contains several conserved domains in common with Type IV restriction enzyme SauUSI, which is known to cleave methylated cytosine nucleotides [28].

If the improved transfer frequencies when using sExpress compared to CA434 were solely due to the circumvention of the Type IV restriction system then it would be expected that transfer frequencies from CA434 into the *C. sporogenes* ΔCLSPO_c06750 strain should be equivalent to those from sExpress into the wild type. This is due to the fact that sExpress should only prove beneficial due to the avoidance of restriction from the *C. sporogenes* NCIMB10696 Type IV restriction system which will attack DNA from the CA434 donor but not from sExpress. If CLSPO_c06750 encodes the only Type IV restriction enzyme in the *C. sporogenes* genome then the advantage sExpress has over CA434 for this strain should be removed. The △CLSPO_c06750 *C. sporogenes* strain was created by first generating a ClosTron mutant which was subsequently converted to a clean deletion using CRISPR/Cas9 genome editing. Although the ClosTron mutants presented increased conjugation efficiencies when compared to the wild type strain (data not shown), a clean deletion of the gene was desirable to rule out the possibility of this phenomenon being due to polar effects of the insertion. To achieve this, an in frame deletion of the CLSPO_c06750 gene was obtained using a CRISPR/Cas9 essentially using a previously described system [23]. In this case the ClosTron insertion of the previously created CLSPO_c06750-1241a:CT strain was the target of the sgRNA converting the ClosTron mutant to an in frame deletion mutant. The construction of mutants is detailed in the materials and methods section.

The transfer efficiency of CA434 into the △CLSPO_c06750 strain is similar to the transfer rate of sExpress into wild type *C. sporogenes* NCIMB 10696 (Fig. 3). sExpress offers no advantage over CA434 in the Type IV knockout strain implying that the advantage it offers in the WT strain is solely due to the lack of Dcm methylation.

### 2.7 sExpress in a PCR ribotype 027 *C. difficile* strain, R20291

PCR Ribotype (RT) 027 strains have historically been associated with more severe disease, increased mortality, higher relapse rates and increased resistance to fluoroquinolone antibiotics [1]. As a consequence, they are frequently referred to as ‘hyper-virulent’ [9]. RT 027 strains first came to prominence at the turn of this century in North America where they were responsible for dramatic increases in *C. difficile* infection (CDI) rates in Canada [1]. Shortly thereafter, their arrival in the UK was signalled by two serious CDI outbreaks at Stoke Mandeville hospital in 2003 and again in 2005 [29]. During these two outbreaks, a total of 498 cases were reported with 172 deaths [29]. The strain responsible was R20291, which has gone on to become a model for RT 027 strains [30].

**Fig. 4:**
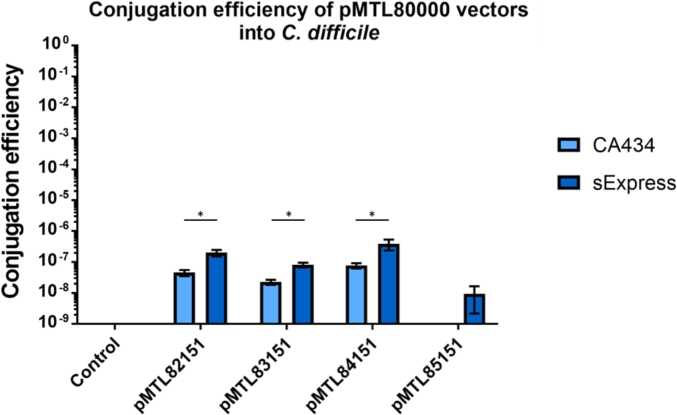
Comparative efficiencies of conjugative plasmid transfer of various vectors from the *E. coli* donors CA434 and sExpress into the recipient strain *C. difficile* R20291. The indicated plasmids, along with a control pMTL80000 plasmid lacking a functional Gram-positive replicon, were transferred from either donor using the conjugation method outlined in the materials and methods section. Conjugation efficiency was calculated as the proportion of putative transconjugant thiamphenicol resistant colonies divided by the CFU of recipient cells. Bars represent the means of three separate conjugations with error bars representing standard deviation. Statistical significance was determined using multiple unpaired t-tests, with one asterisk denoting a (p < 0.05 *, n = 3).

In common with other RT 027 strains, the frequency of plasmid transfer into R20291 in conjugations with CA434 is low. Given the presence of predicted Type IV restriction systems it was hypothesised that the sExpress conjugal donor strain could increase frequencies of conjugation as previously observed in the other two clostridial species examined in this study. Fig. 4 demonstrates that sExpress offers significantly increased frequency of conjugative plasmid transfer of a range of vectors, based on different clostridial replicons, into *C. difficile* R20291 compared to the use of CA434 as the donor. In the one instance, pMTL85151 (which carries the pIM13 Gram-positive replicon), transfer could not be demonstrated using CA434 as the donor strain. In contrast, using sExpress as the donor, R20291 transconjugants were reproducibly obtained.

**Fig. 5:**
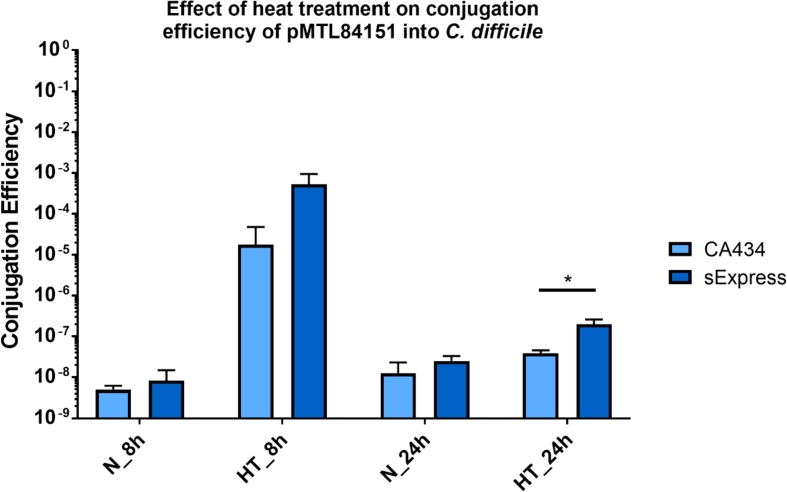
Determination of conjugation efficiency into *C. difficile* R20291 using the Kirk et al. protocol. *E. coli* donors CA434 or Sexpress were used to transfer plasmid pMTL84151 into *C. difficile* R20291 following 8 or 24 h (h) incubation on mating plates, with (HT) or without (N) prior heat-treatment of recipient R20291 cells. This experiment utilised the Kirk et al. [31]method of conjugative transfer. Briefly, *C. difficile* R20291 recipient cells were grown overnight in TY broth, from which 200 μl aliquots were taken and either heat-treated at 52 °C for 5 min or not, before being used to resuspend 1 ml of *E. coli* donor cell pellets. The resulting suspension was spotted onto antibiotic-free Brain Heart Infusion (BHI) agar and incubated anaerobically for 8 or 24 h. Growth on mating plates was then harvested and diluted in TY broth and plated onto BHI agar plates supplemented with d-cycloserine, cefoxitin and thiamphenicol where appropriate. Conjugation efficiency was calculated as the ratio of thiamphenicol resistant CFU to total *C. difficile* CFU. Bars represent the means of three separate conjugations with error bars representing standard deviation. The two donor strains were compared for each condition and statistical significance was determined using multiple unpaired t-tests, with one asterisk denoting a (p < 0.05 *, n = 3). The comparison of sExpress and CA434 with heat shock and 8 h mating plate time had a p value of 0.105.

Exposing the recipient *C. difficile* cells to heat shock prior to mixing with donor *E. coli* cells has previously been shown to increase conjugation efficiencies [31]. One possible explaination for the increase was suggested to be due to the inactivation of *C. difficile* restriction systems. To investigate this idea the published heat shock protocol was followed using both CA434 and sExpress as donors (Fig. 5). Our results show that the advantage of sExpress as a donor was retained even with heat shock, suggesting that it is not the Type IV restriction system which sExpress circumvents which is being inactivated by the heat shock, though other restriction systems may be.

## 3 Discussion

In the current study we have demonstrated that *E. coli* sExpress represents a superior conjugal donor for the transfer of plasmids to the clostridial recipient strains *C. autoethanogenum* DSM 10061, *C. sporogenes* NCIMB 10696 and *C. difficile* R20291 compared to the *E.coli* donor strain CA434. For *C. autoethanogenum* DSM 10061 it is worth noting that of the ten separate conjugations performed only six produced any transconjugants when using CA434 as the conjugal donor. Historically, this lack of reproducible plasmid transfer has inevitably slowed progress in strategies reliant on genetic modification of the strain, a situation exacerbated by the extended period of time required for the development of transconjugant colonies, which typically arise on day 6 of the process. The unreliability of conjugative transfer using CA434 as the donor has necessitated setting up multiple conjugation replicates. These inefficiencies are compounded by further decreases in efficiencies when using plasmids larger than pMTL83151 or when using a more defective Gram-positive replicons than pCB102 as is preferred when genome editing following the Allele-Coupled Exchange (ACE) procedure [32,33].

A knockout strain for CLAU_0514; which encodes a putative Type IIG fused restriction-modification enzyme was generated via CRISPR-Cas9. The resultant strain exhibited one order of magnitude higher conjugation efficiencies over the wild type providing firm evidence for the relevance of this enzyme to the restriction barrier in *C. autoethanogenum* DSM 10061. This strain could be useful for studies for which a high DNA transfer rate is required. More benefit was derived from the generation of the sExpress donor strain, however, which produced approximately 600 times the number of transconjugants per conjugation than CA434 (Fig. 2) and can be used with the wild type strain of *C. autoethanogenum* DSM 10061.

The logic behind the creation of sExpress was to avoid Type IV restriction systems which target DNA methylated at CCWGG sites. There are numerous genetic differences between CA434 and sExpress other than the presence of *dcm* in CA434. sExpress is a B strain derivative whereas CA434 is predominately based on K-12 strain (96.9%) with only the remaining portion being derived from *E. coli* B [34]. The hypothesis that the *dcm* status of the donor strain is the salient difference in terms of conjugation efficiency could be clearly examined by generating a clean deletion of *dcm* in the CA434 strain. However, it seems likely that the *dcm* status is the most relevant factor of this strain since in the Type IV knockout strain of *C. sporogenes* NCIMB 10696, sExpress exhibited no significant difference in conjugation efficiency compared to CA434; suggesting that the only benefit sExpress did have was via avoidance of the Type IV restriction system. This result implies that *C. sporogenes* CLSPO_c06750 encodes a Type IV, cytosine-acting restriction system and that no other cytosine-acting Type IV restriction systems are present in the *C. sporogenes* NCIMB 10696 genome which target Dcm methylated DNA. It is possible that a circumvention of the remaining restriction systems of *C. sporogenes* NCIMB10696 will improve DNA transfer further. In particular, the Type I restriction system encoded by the loci CLSP_c17010-CLSPO_c17030 may represent a significant restriction barrier which could be overcome by the generation of a knockout strain for this region, or by the mimicking of the methylation pattern encoded by the methyltransferase of the complex.

The importance of Dam/Dcm methylation may not be due to the effect of Type IV restriction systems in all cases. In *Bacillus anthracis* it was found that knocking out all of the putative Type IV systems improved transformation frequency with Dam + Dcm + DNA and yet Dcm- Dam- DNA still transformed with greater efficiency [11]. With no more obvious Type IV targets it was thought more likely that there must be an alternative mechanism by which methylated DNA is less able to transform *B. anthracis*. This phenomenon is also documented in both Dam-strains of *E. coli* [35] and *Salmonella enterica* [36] which are easily transformed by unmethylated DNA and suffer efficiency losses with Dam methylated DNA. Plasmids with Dam + Dcm + methylation entering a recipient strain which lacks *dam* and *dcm* methyltransferases will form hemimethylated products after their first replication cycle. Hemimethylated DNA has been shown to be a poor substrate for DNA replication in *dam- E. coli* strains where hemimethylated plasmids accumulate [35]. Perhaps the efficiency gains by avoiding Dcm methylation are not entirely due to avoidance of Type IV systems but due to a more suitable substrate for further replication cycles after the first replication event. In addition, a *dam-*donor strain could confer benefit even to strains which lack any adenine-directed Type IV systems. The creation of such a strain would be of interest to help deduce the specificity of the Type IV systems of these species. One downside of *dam- E. coli* strains is that they exhibit significantly higher mutation rates due to the dependence of the mismatch repair mechanism on adenine-methylated DNA [37].

Improved DNA transfer rates will facilitate genetic studies in *Clostridium* species. There are species of interest for which no established DNA transfer protocol exist such as *C. carboxidivorans* and *C. scindens*, and there are many more species where mutant generation procedures would benefit from an improved DNA transfer rate. For instance an I-SceI mediated gene modification strategy was limited by transformation rates and optimization of this step was explicity called for [38]. The restriction systems of an organism can be predicted from a genome sequence. The online resource REBASE provides information on the restriction systems of many organisms allowing a strategy to circumvent these systems to be produced [21]. Single Molecule Real Time sequencing techniques allow the identification of modified bases and hence the identification of motifs which are methylated for a given genome. This information is invaluable in the analysis of the restriction systems of an organism and is available in the REBASE databases in many cases. sExpress has proven to be a useful donor strain in the three organisms *C. autoethanogenum* DSM 10061*, C. sporogenes* NCIMB 10696 and *C. difficile* R20291. It is likely that the benefits of using this donor over CA434 will extend to many similar species which have cytosine directed Type IV restriction systems. REBASE can be used to predict the presence of these Type IV systems, in cases where SMRT sequencing data has revealed DNA modifications the relevance of various restriction systems is especially notable. In cases where DNA transfer is especially difficult to achieve it may be necessary to completely mimic the DNA modification pattern of the host strain. This can be achieved by cloning the host methyltransferase genes into a conjugal or DNA preparation shuttle strain.

The method used to generate the *C. sporogenes* ΔCLSPO_c06750 strain can be a valuable tool to generate in frame deletions of any gene previously targeted with ClosTron. One of the main constraints of the CRISPR-Cas9 technology is that it strongly depends on an efficient sgRNA. This was demonstrated by the fact that of the three seed sequences tested in this study only sgRNA 3 (5′-TGGATATTCACCGAACACTA-3′) provided sufficient counter-selective pressure to allow the isolation of in-frame deletion mutants. This sgRNA targets the *ermB* gene from the ClosTron insert, meaning it can efficiently target any ClosTron-containing gene. Given the prevalence of ClosTron mutants described in the literature and the common desire to convert a ClosTron mutant into a clean deletion mutant, it is hoped that the description of this effective guide region will prove of benefit to researchers wanting to undertake this task.

## 4 Materials and methods

### 4.1 Growth of bacterial strains

*Escherichia coli* species were routinely cultured in LB broth with appropriate antibiotic selection unless otherwise stated. Broth cultures were propagated in a shaking incubator at 37 °C under 225 RPM, while agar plates were incubated statically at 37 °C. *Clostridium autoethanogenum* DSM 10061 was propagated in YTF medium (10 g L^−1^ yeast extract, 16 g L^−1^ tryptone, 10 g L^−1^ fructose, 0.2 g L^−1^ sodium chloride, pH 5.8–6). YTF was supplemented with appropriate antibiotic selection, and incubated in a Don Whitley anaerobic workstation under an atmosphere of 80% nitrogen, 10% carbon dioxide, 10% hydrogen at 37 °C. Additional clostridial strains were also incubated under these anaerobic conditions. *Clostridium difficile* strains were propagated on brain heart infusion medium supplemented with 5 g.L^−1^ yeast extract and 0.1% l-cysteine (BHIS). *Clostridium sporogenes* strains were propagated on TYG medium (20 g L^−1^ trypticase, 5 g L^−1^ peptone, 1 g L^−1^ glucose, 5 g L^−1^ yeast extract and 1 g L^−1^ cysteine-HCl). Growth media was supplemented with antibiotics at the following working concentrations; chloramphenicol (25 μg ml^−1^), thiamphenicol (15 μg ml^−1^), d-cycloserine (250 μg ml^−1^), spectinomycin (100 μg ml^−1^), kanamycin 50  μg ml^−1^, tetracycline (10  μg ml^−1^). cefoxitin (10  μg ml^−1^).

### 4.2 Generation of the sExpress conjugal donor

A novel conjugal donor strain was created via conjugal transfer of the plasmid R702 from CA434 into the *E. coli* strain NEB Express. The genotype of NEB Express is: *fhuA2 [lon] ompT gal sulA11 R(mcr-73*:*miniTn10*--*TetS)2 [dcm] R(zgb-210*:*Tn10*--*TetS) endA1 Δ(mcrC-mrr)114*:*IS10*. Two 5 ml LB broth cultures were inoculated with donor (CA434) and recipient (NEB Express) strains respectively from glycerol stocks, and following overnight incubation were sub-cultured into fresh medium and grown to an OD_600_ of approximately 0.2. Donor and recipient cultures were incubated statically together for one hour at 37 °C, before being transferred to M9 salts minimal medium lacking leucine and proline but supplemented with all other required amino acids at 100 mg ml^−1^ to select against the donor CA434 and supplemented with tetracycline to select for R702. Transconjugants were re-streaked on the same selective media and screened for the presence of R702 and identity as NEB Express, through acquisition of antibiotic resistance and absence of auxotrophies, respectively. All transconjugants screened were shown to be NEB Express cells harbouring R702 and this strain was subsequently named sExpress.

### 4.3 *C. autoethanogenum* conjugations

A *C. autoethanogenum* starter culture was initiated from glycerol stocks in non-selective YTF broth over a period of 72 h, until late exponential phase. Prior to conjugation the culture was then sub-cultured into fresh YTF broth (5 ml) and incubated for 16–20 h, until an OD_600_ of approximately 0.1–0.2 was achieved. The donor strain harbouring the shuttle vector was inoculated into LB broth supplemented with appropriate antibiotic selection for the marker as present on the transferring vector well as kanamycin to continue to select for R702, and incubated for 14–16 h. Approximately four hours prior to conjugation the donor strain was sub-cultured into fresh LB broth containing antibiotic selection and incubated until an OD_600_ of between 0.2 and 0.4 was reached. Subsequently 1 ml of donor culture was aliquoted into a 1.5 ml microcentrifuge tube using a wide bore pipette, and centrifuged at 3000 *g* for three minutes. The supernatant was aspirated and discarded, and the cell pellet gently re-suspended in 500 μl of phosphate buffered saline (PBS) by gentle flicking and inversion. The centrifugation step was repeated, the supernatant aspirated and discarded, and the cell pellet transferred to the anaerobic workstation. All subsequent steps were performed under anaerobic conditions. The donor cell pellet was gently re-suspended in 200 μl of recipient cell culture, again through careful flicking and inversion rather than by repeated aspiration. The combined cultures were then transferred to an anaerobic YTF agar plate without antibiotic supplementation, spread using a wedge shaped spreader, and incubated for approximately 20 h. Subsequently, 500 μl of anaerobic PBS was used to flood the surface of the agar plate, and a wedge-shaped spreader used to gently dislodge and re-suspend the bacterial growth into the liquid. The resultant bacterial slurry was collected by aspiration using a pipette, and transferred to selective YTF agar plates supplemented with appropriate antibiotic selection for the marker present on the shuttle vector and d-cycloserine was used to counter-select the *E. coli* donor. Transconjugant colonies of the recipient strain should become visible following approximately 72 h of incubation, with growth rate dependant on the Gram-positive replicon present on the shuttle vector.

### 4.4 DNA manipulations

Genomic DNA serving as PCR template was prepared using genomic DNA extraction kits (Sigma Aldrich). Plasmid DNA was prepared using Mini-prep kits (Sigma Aldrich). PCRs were carried out using Q5 polymerase (NEB) or dreamtaq (Thermo Fisher) for screening PCRs. Oligonucleotides were synthesised by Sigma-Aldrich or Eurofins. Sanger sequencing was outsourced to Source Bioscience (United Kingdom) or Eurofins (Germany). HiFi reactions were carried out using NEBuilder® HiFi DNA Assembly Master Mix (NEB).

### 4.5 *C. sporogenes* conjugations

Conjugations into *C. sporogenes* NCIMB 10696 were very similar to *C. autoethanogenum*, with the following modifications: *C. sporogenes* strains were grown for 14–16 h in TYG broth. Before conjugation, the strains were sub-cultured into fresh TYG broth and incubated for 3–4 h until an OD_600_ of 0.7–0.8 was achieved. Mating cells were incubated for approximately 24 h and transconjugant colonies were visible after approximately 20 h.

### 4.6 *C. difficile* conjugations

Conjugations into *C. difficile* R20291 were performed as for *C. autoethanogenum* and *C. sporogenes* with several modifications: overnight cultures of donor strains were used without subculturing, mating cells were incubated for eight hours and cefoxitin was used alongside d-cycloserine on transconjugant selection plates. Heat shocked conjugations were performed as detailed in Ref. [31] which involved a 2 min heat shock at 52 °C prior to mixing with the donor *E. coli* culture.

### 4.7 Generation of *C. autoethanogenum* ΔCLAU_0514

The CLAU_0514 gene was targeted using CRISPR cas9 gene editing techniques, whereby a synthetic guide targeting a unique sequence contained within the gene in question is supplied on a vector conferring thiamphenicol resistance, along with a nucleolytically active cas9 from *Streptococcus pyogenes* and a homologous repair cassette, a knockout technique previously shown to be effective in this organism [23]. The homology cassette was composed of 1 kb homology arms flanking CLAU_0514, assembled by overlap extension PCR using the oligonucleotides CLAU_0514_3’_F, CLAU_0514_3’_R, CLAU_0514_5’_F, and CLAU_0514_5’_R, and were incorporated into a modified version of the CRISPR cas9 vector through traditional restriction ligation cloning methods. The synthetic guide targeting sequence was introduced using the NEB HiFi Assembly cloning kit to incorporate a single stranded oligonucleotide (Sigma Aldrich) into the linearised CRISPR cas9 vector, yielding the plasmid pMTL43151-CLAU_0514. This plasmid was verified using Sanger sequencing, transformed into the conjugative donor strain sExpress, and conjugated into *C. autoethanogenum*. Resultant colonies which harboured resistance to thiamphenicol were screened at the CLAU_0514 locus for the intended in-frame deletion, and the PCR product sequenced to confirm the nature of the deletion. Following sequence confirmation, plasmid loss was achieved through serial passage in the absence of antibiotic selection, confirmed through replica plating, and the resultant strain stored at −80 °C.

### 4.8 Generation of the *C. sporogenes* △CLSPO_c06750 strain

The CLSPO_c06750 gene was targeted using ClosTron [39]. This approach allows the creation of mutants by the insertion of a group II intron containing a retrotransposition-activated marker into a target gene, causing loss of function by disruption of the gene. The intron was re-targeted using a computer algorithm available on the website www.clostron.com [39]. A pMTL007 vector containing the re-targeted intron (pMTL007:Csp-CLSPO_c06750-1241a) was obtained from DNA2.0 Inc. The vector was electroporated into *E. coli* CA434 and then conjugated into *C. sporogenes* NCIMB 10696 as previously described. Transconjugants were selected on TYG plates containing d-cycloserine and thiamphenicol, because the pMTL007 contains the *catP* gene in the backbone. Cells containing the intron were selected on TYG plates containing d-cycloserine and erythromycin since the *ermB* gene was used as the retrotransposition-activated marker. The insertion of the intron into the CLSPO_c06750 gene was screened by PCR and confirmed by sequencing. The mutant strain was named *C. sporogenes*- CLSPO_c06750-1241a:CT.

Three different sgRNA seeds targeting the ClosTron insert were designed using Benchling software (www.benchling.com) (Table 1). sgRNA 1 and sgRNA 3 target the *ermB* gene (retrotransposition-activated marker), and sgRNA 2 targets the beginning of the ClosTron insert. sgRNA cassettes including P_*araE*_, the seed and the *fdx* terminator were assembled by overlap extension PCR.

**Table 1:** sgRNA seed regions used to target the ClosTron insertion.

sgRNA seed	Sequence (5’ → 3′)
sgRNA 1	GCAATTGCTTAAGCTGCCAG
sgRNA 2	TGCTCTGTTCCCGTATCAGC
**sgRNA 3**	**TGGATATTCACCGAACACTA**

Bold = only sgRNA that proved effective.

Homology arms (1 Kb each) flanking the CLSPO_c06750 gene were also designed and assembled by overlap extension PCR. These included the first two and last three codons of the CLSPO_c06750 gene.

The sgRNA and homology arm cassettes were incorporated into a modified version of the CRISPR cas9 vector described previously through traditional restriction ligation cloning methods, yielding plasmids pMTL43151_CLSPO_c06750_sgRNA1, pMTL43151_CLSPO_c06750_sgRNA2, and pMTL43151_CLSPO_c06750_sgRNA3 which were sequence confirmed with Sanger sequencing. The vectors were electroporated into *E. coli* CA434 and then conjugated into *C. sporogenes*- CLSPO_c06750-1241a:CT. Transconjugants were selected on TYG plates containing d-cycloserine and thiamphenicol. The in-frame deletion of the CLSPO_c06750 gene (obtained only with sgRNA 3) was screened by PCR and confirmed by sequencing. The mutant strain was named *C. sporogenes* ΔCLSPO_c06750.

### 4.9 Strain availability

sExpress is a deriviative of NEB Express (NEB catalog #C2523) which means it is subject to commercial restrictions. Briefly, licensing is not required for internal research and development but if it is used for the production of or as part of a commercial product then commercial restrictions apply and a licensing agreement is required. For more information about commercial rights, please contact NEB's Global Business Development team at gbd@neb.com.

## Author contributions

CW, CMH and NPM planned the study. CW, CMH and PR undertook the experimental work in *C. autoethanogenum*, RMR in *C. sporogenes* and PI in *C. difficile.* Data analysis was undertaken by all authors. CW, CMH and NPM drafted the manuscript, all authors contributed to the final version.

## Competing interests

The authors declare that they have no competing interests.

## References

[1] Kuijper E.J., Coignard B., Tüll P. (2006). Emergence of Clostridium difficile-associated disease in North America and Europe. Clin. Microbiol. Infect..

[2] Humphreys C.M., Minton N.P. (2018). Advances in metabolic engineering in the microbial production of fuels and chemicals from C1 gas. Curr. Opin. Biotechnol..

[3] Heap J.T., Theys J., Ehsaan M., Kubiak A.M., Dubois L., Paesmans K., Van Mellaert L., Knox R., Kuehne S.A., Lambin P., Minton N.P. (2014). Spores of Clostridium engineered for clinical efficacy and safety cause regression and cure of tumors in vivo. Oncotarget.

[4] Minton N.P., Ehsaan M., Humphreys C.M., Little G.T., Baker J., Henstra A.M., Liew F., Kelly M.L., Sheng L., Schwarz K., Zhang Y. (2016). A roadmap for gene system development in Clostridium. Anaerobe.

[5] Liew F., Henstra A.M., Kӧpke M., Winzer K., Simpson S.D., Minton N.P. (2017). Metabolic engineering of Clostridium autoethanogenum for selective alcohol production. Metab. Eng..

[6] Cooksley C.M., Davis I.J., Winzer K., Chan W.C., Peck M.W., Minton N.P. (2010). Regulation of neurotoxin production and sporulation by a putative agrBD signaling system in proteolytic Clostridium botulinum. Appl. Environ. Microbiol..

[7] Theys J., Pennington O., Dubois L., Anlezark G., Vaughan T., Mengesha A., Landuyt W., Anné J., Burke P.J., Dûrre P., Wouters B.G., Minton N.P., Lambin P. (2006). Repeated cycles of Clostridium-directed enzyme prodrug therapy result in sustained antitumour effects in vivo. Br. J. Canc..

[8] Cartman S.T., Kelly M.L., Heeg D., Heap J.T., Minton N.P. (2012). Precise manipulation of the Clostridium difficile chromosome reveals a lack of association between the tcdC genotype and toxin production. Appl. Environ. Microbiol..

[9] Cartman S.T., Heap J.T., Kuehne S.A., Cockayne A., Minton N.P. (2010). The emergence of ‘hypervirulence’ in Clostridium difficile. Int J Med Microbiol.

[10] Purdy D., O'Keeffe T.A., Elmore M., Herbert M., McLeod A., Bokori-Brown M., Ostrowski A., Minton N.P. (2002). Conjugative transfer of clostridial shuttle vectors from [i]Escherichia coli[/i] to [i]Clostridium difficile[/i] through circumvention of the restriction barrier. Mol. Microbiol..

[11] Sitaraman R., Leppla S.H. (2012). Methylation-dependent DNA restriction in Bacillus anthracis. Gene.

[12] González-Cerón G., Miranda-Olivares O.J., Servín-González L. (2009). Characterization of the methyl-specific restriction system of Streptomyces coelicolor A3(2) and of the role played by laterally acquired nucleases. FEMS Microbiol. Lett..

[13] Kieser T., Hopwood D.A. (1991). Genetic manipulation of Streptomyces: integrating vectors and gene replacement. Methods Enzymol..

[14] Macalusot A., Mettus A.-M. (1991). Efficient transformation of Bacillus thuringiensis requires nonmethylated plasmid DNA. J. Bacteriol..

[15] Sladek T.L., Nowak J.A., Maniloff J. (1986). Mycoplasma restriction: identification of a new type of restriction specificity for DNA containing 5-methylcytosine. J. Bacteriol..

[16] Macneil D.J. (1988). Characterization of a unique methyl-specific restriction system in Streptomyces avermitilis. J. Bacteriol..

[17] Spath K., Heinl S., Grabherr R. (2012). Direct cloning in Lactobacillus plantarum: electroporation with non-methylated plasmid DNA enhances transformation efficiency and makes shuttle vectors obsolete. Microb. Cell Factories.

[18] Guss A.M., Olson D.G., Caiazza N.C., Lynd L.R. (2012). Dcm methylation is detrimental to plasmid transformation in clostridium thermocellum. Biotechnol. Biofuels.

[19] Banerjee A., Leang C., Ueki T., Nevin K.P., Lovley D.R. (2014). Lactose-inducible system for metabolic engineering of clostridium ljungdahlii. Appl. Environ. Microbiol..

[20] Kolek J., Sedlar K., Provaznik I., Patakova P. (2016). Dam and Dcm methylations prevent gene transfer into Clostridium pasteurianum NRRL B-598: development of methods for electrotransformation, conjugation, and sonoporation. Biotechnol. Biofuels.

[21] Roberts R.J., Vincze T., Posfai J., Macelis D. (2015). REBASE-a database for DNA restriction and modification: enzymes, genes and genomes. Nucleic Acids Res..

[22] Krüger T., Wild C., Noyer-Weidner M. (1995). McrB: a prokaryotic protein specifically recognizing DNA containing modified cytosine residues. EMBO J..

[23] Ingle P., Groothuis D., Rowe P., Huang H., Cockayne A., Kuehne S.A., Jiang W., Gu Y., Humphreys C.M., Minton N.P. (2019). Generation of a fully erythromycin-sensitive strain of *Clostridioides difficile* using a novel CRISPR-Cas9 genome editing system. Sci Rep..

[24] Heap J.T., Pennington O.J., Cartman S.T., Minton N.P. (2009). A modular system for Clostridium shuttle plasmids. J. Microbiol. Methods.

[25] Kubiak A.M., Poehlein A., Budd P., Kuehne S.A., Winzer K., Theys J., Lambin P., Daniel R., Minton N.P. (2015). Complete genome sequence of the nonpathogenic soil-dwelling bacterium *Clostridium sporogenes* strain NCIMB 10696. Genome Announc..

[26] Wang H.H., Isaacs F.J., Carr P.A., Sun Z.Z., Xu G., Forest C.R., Church G.M. (2009). Programming cells by multiplex genome engineering and accelerated evolution. Nature.

[27] Langridge G.C., Phan M.D., Turner D.J., Perkins T.T., Parts L., Haase J., Charles I., Maskell D.J., Peters S.E., Dougan G., Wain J., Parkhill J., Turner A.K. (2009). Simultaneous assay of every Salmonella Typhi gene using one million transposon mutants. Genome Res..

[28] Xu S.Y., Corvaglia A.R., Chan S.H., Zheng Y., Linder P. (2011). A type IV modification-dependent restriction enzyme SauUSI from Staphylococcus aureus subsp. aureus USA300. Nucleic Acids Res..

[29] Commission H. (2006). Investigation into Outbreaks of Clostridium difficile at Stoke Mandeville Hospital.

[30] Stabler R.A., He M., Dawson L., Martin M., Valiente E., Corton C., Lawley T.D., Sebaihia M., Quail M.A., Rose G., Gerding D.N., Gibert M., Popoff M.R., Parkhill J., Dougan G., Wren B.W. (2009). Comparative genome and phenotypic analysis of Clostridium difficile 027 strains provides insight into the evolution of a hypervirulent bacterium. Genome Biol..

[31] Kirk J.A., Fagan R.P. (2016). Heat shock increases conjugation efficiency in Clostridium difficile. Anaerobe.

[32] Al-Hinai M.A., Fast A.G., Papoutsakis E.T. (2012). Novel system for efficient isolation of clostridium double-crossover allelic exchange mutants enabling markerless chromosomal gene deletions and DNA integration. Appl. Environ. Microbiol..

[33] Ng Y.K., Ehsaan M., Philip S., Collery M.M., Janoir C., Collignon A., Cartman S.T., Minton N.P. (2013). Expanding the repertoire of gene tools for precise manipulation of the Clostridium difficile genome: allelic exchange using pyrE Alleles. PLoS One.

[34] Jeong H., Sim Y.M., Kim H.J., Lee S.J. (2017). Unveiling the hybrid genome structure of Escherichia coli RR1 (HB101 RecA+). Front. Microbiol..

[35] Russell D.W., Zinder N.D. (1987). Hemimethylation prevents DNA replication in E. coli. Cell.

[36] Aloui A., Chatty A., El May A., Landoulsi A. (2007). The effect of methylation on DNA replication in Salmonella enterica serovar typhimurium. Comptes Rendus Biol..

[37] Marinus M.G. (2010). DNA methylation and mutator genes in Escherichia coli K-12. Mutat. Res. Rev. Mutat. Res..

[38] Zhang N., Shao L., Jiang Y., Gu Y., Li Q., Liu J., Jiang W., Yang S. (2015). I-SceI-mediated scarless gene modification via allelic exchange in Clostridium. J. Microbiol. Methods.

[39] Heap J.T., Pennington O.J., Cartman S.T., Carter G.P., Minton N.P. (2007). The ClosTron: a universal gene knock-out system for the genus Clostridium. J. Microbiol. Methods.

